# Hyperleukocytosis in Pediatric Patients with Acute Lymphoblastic Leukemia: Demographic and Clinical Characteristics

**DOI:** 10.3390/jcm13175185

**Published:** 2024-09-01

**Authors:** Małgorzata Monika Mitura-Lesiuk, Maciej Dubaj, Aleksandra Dembowska, Karol Bigosiński, Mateusz Raniewicz

**Affiliations:** 1 Department of Paediatric Haematology, Oncology, and Transplantology, Medical University of Lublin, 20-093 Lublin, Poland; 2 Student Scientific Society of the Department of Paediatric Haematology, Oncology, and Transplantology, Medical University of Lublin, 20-093 Lublin, Poland; 56477@student.umlub.pl (M.D.); 55204@student.umlub.pl (A.D.); 56632@student.umlub.pl (K.B.); 56580@student.umlub.pl (M.R.)

**Keywords:** hyperleukocytosis, acute lymphoblastic leukemia, children, risk factor, prognosis

## Abstract

**Background**: Hyperleukocytosis, defined as a total leukocyte count of more than 50,000/mm^3^ leukocytes, occurs in almost one in five children with acute lymphoblastic leukemia (ALL). It represents an unfavorable prognostic factor in this disease. The aim of the following study was to describe demographic and clinical features in patients with hyperleukocytosis and their relationship with leukocyte count. **Methods**: We retrospectively analyzed the available medical data of patients with ALL diagnosed and treated at the University Children’s Hospital in Lublin between 2017 and 2024. **Results**: Of the 97 patients, 10 (10.3%) had hyperleukocytosis. They were significantly more likely to be older boys diagnosed with T-ALL. The group with hyperleukocytosis had a higher mortality rate. The presence of hyperleukocytosis also correlated with the presence of petechiae, thrombocyte and neutrophil counts, and LDH activity. Patients with hyperleukocytosis also experienced a higher incidence of infections as a complication of therapy as leukocyte counts increased. **Conclusions**: Hyperleukocytosis, although rare, is an important factor in the course of ALL, both clinically and prognostically.

## 1. Introduction

### 1.1. Acute Lymphoblastic Leukemia

Acute lymphoblastic leukemia (ALL) is a group of hematological malignancies that are characterized by the abnormal proliferation of immature lymphoid cells. It is the most frequently diagnosed cancer in childhood (80% of acute leukemia cases), and its cure rate is as high as 80%. Leukemias in children can be divided into those originating from B or T cell precursors, with the former accounting for 85% of cases. The peak incidence occurs between the ages of 1 and 4 but decreases rapidly in the following years [1]. There is currently no evidence for clear causes of ALL. However, several risk factors have been identified that have a real impact on the occurrence of the disease. Environmental factors include exposure to ionizing radiation, the impact of chemical compounds, and infections (HTLV-1). Regarding genetic factors, childhood leukemia often occurs in patients with t(9;22) translocation, Down’s syndrome, Bloom syndrome, or neurofibromatosis. ALL is characterized by a very wide and non-specific group of symptoms. It may start with fever, weakness, bone, and joint pain. Due to anemia, patients experience lower exercise tolerance, pallor, and shortness of breath. Thrombocytopenia may manifest itself through bleeding gums and noses and the presence of signs of bleeding diathesis (petechiae, run-ups, and hemorrhages). Neutropenia may be associated with features of recurrent infection that do not respond to standard antibiotic therapy. Another phenomenon observed in the blood count of peripheral blood, which may be life-threatening for patients with ALL, is hyperleukocytosis and the resulting clinical consequences [2].

### 1.2. Hyperleukocytosis

Hyperleukocytosis is defined as a white blood cell count above 50,000/mm^3^. It occurs in 10.2 to 19.2% of children with ALL. It is a factor that causes a significantly higher risk of death in relation to cases without hyperleukocytosis [3]. Patients with hyperleukocytosis have a 3-to-5 times higher risk of leukostasis and conditions secondary to it, such as intracranial bleeding, seizures, coagulopathy, renal failure, metabolic disorders related to tumor lysis, and respiratory issues [3]. The treatment of symptomatic hyperleukocytosis includes various methods of leukoreduction, which include intravenous hydration, the initiation of chemotherapy, hydroxyurea treatment, and leukapheresis. The last one, in particular, is a quick and effective method for reducing the number of leukocytes in the peripheral blood [4]. Hyperleukocytosis of the range 50,000- 100,000/mm^3^ remains most often asymptomatic in patients with ALL but may give early signs of leukostasis in children with acute myeloblastic leukemia (AML). In contrast, only results based on the order of 100,000 cells/mm3 are associated with the appearance of the aforementioned symptoms and clinical conditions in patients with ALL [5,6]. In ALL, hyperleukocytosis is significantly more often associated with its T-cell subtype (T-ALL). Characteristic genetic alterations in hyperleukocytosis are *KMT2A* mutations (including rearrangements with *AFF1* and *MLLT1/3* genes), *ETV6-RUNX1* rearrangement, the mutation of genes *LYL1*, *DNMT3A*, *FLT3*, *NPM1*, and sometimes the presence of the *BCR-ABL* fusion gene [5,7,8,9]. Other hematological diseases in which hyperleukocytosis can occur are chronic myeloid leukemia (CML) and chronic myelomonocytic leukemia (CMML) [5]. Researchers agree that hyperleukocytosis is one of the risk factors for poor prognosis in ALL. They point out that both overall survival and event-free survival rates are lower in children with hyperleukocytosis, especially in cases of extreme hyperleukocytosis >200,000/mm^3^ [9,10,11]. The problem of this phenomenon is so significant that each week of delayed diagnosis of the disease results in an increase in mortality by as much as 40% [5,7]. Unfortunately, the topic of hyperleukocytosis among pediatric patients has not been fully studied and clarified. Data are available in a few publications, more often on AML than ALL. This is especially true with regard to the epidemiology and impact of hyperleukocytosis on the course and outcome of treatment. There are practically no papers on this topic from the Central and Eastern European regions.

The main aim of this paper was to present demographic and clinical data on hyperleukocytosis among patients with ALL from a single Polish hematooncology center, along with the outcomes and complications of their treatment.

## 2. Materials and Methods

### 2.1. Data Collection

We retrospectively reviewed the medical records of patients hospitalized between January 2017 and February 2024 at the Department of Pediatric Hematology, Oncology and Transplantation at the University Children’s Hospital in Lublin who were diagnosed with acute lymphoblastic leukemia (ALL). Of all these cases, those with hyperleukocytosis on laboratory tests at the time of admission to the department were included in the analysis. Leukocyte counts of WBC = 50,000/mm^3^ were used as the cutoff for hyperleukocytosis [6]. The inclusion criteria were as follows: hyperleukocytosis at the time of enrollment in the clinic and diagnostic management with at least one laboratory result; a definite diagnosis of ALL based on cytogenetic, molecular, and immunophenotypic testing; and residency in the clinic between 2017 and 2024. The exclusion criteria were as follows: unclear disease diagnosis, a final diagnosis other than ALL, insufficient clinical data, or lack of complete patient history. The clinic can only admit patients <18 years of age; thus, age was not a criterion.

### 2.2. Statistical Analysis

For statistical analysis, MedCalc 15.8 software (MedCalc Software, Ostend, Belgium) was used. Before performing the actual statistical calculations, a power analysis was performed. The power of the test was 0.85. Based on this, the size of the study group was determined to be sufficient to consider the results significant. A value of *p* < 0.05 was taken as the criterion for statistical significance. Descriptive statistics were presented by the median and interquartile range (IQR), and nominal categorical variables were presented by the frequency and percentage (%). The D’Agostino–Pearson test was used to analyze normal distribution. Continuous variables were compared using Student’s *t*-test (variables with a normal distribution) or the Mann–Whitney U-test (variables with a distribution different from normal). Categorical variables were compared using Pearson’s chi-square test. To analyze the correlation between leukocytosis values and certain clinical and laboratory characteristics, the rank correlation test was used. Due to the small size of the study group, Spearman’s rank correlation test was crucial for analyzing the relationships and their strength.

## 3. Results

### 3.1. General Characteristics of the Study Group

In the period from January 2017 to February 2024, 97 children with ALL were diagnosed at the clinic. Among them, the majority were women (52.6%), and the median age at diagnosis was 78 (interquartile range [IQR]: 45–143) months. This is equivalent to 6.5 (IQR: 3.75–11.94) years. Most patients were diagnosed with B-ALL (77.3%), with pre-B type predominating (98.67%). During the analyzed period, seven children died due to disease progression in one case due to associated sepsis and, additionally, in one case with hemorrhage into the central nervous system (CNS). The general characteristics of the study group are shown in Table 1.

### 3.2. Comparison of Demographics of Patients with and without Hyperleukocytosis

**Figure 1:**
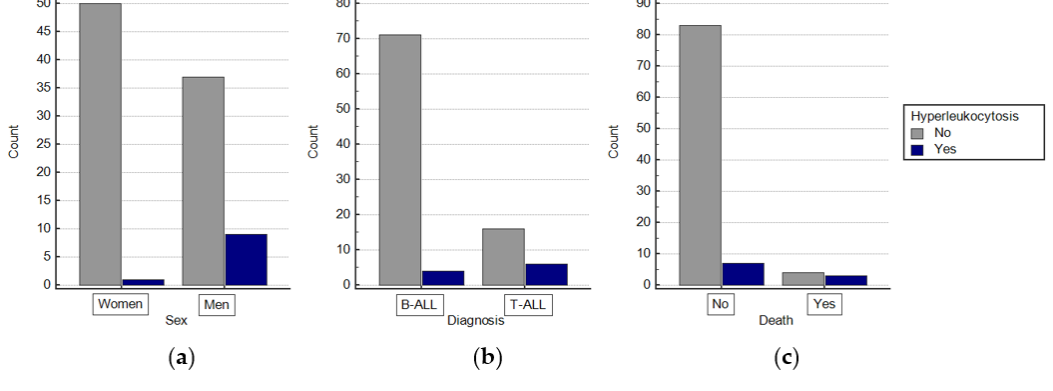
(**a**) Distribution of patients with and without hyperleukocytosis by gender. (**b**) Distribution of patients with and without hyperleukocytosis by type of leukemia; (**c**) distribution of patients with and without hyperleukocytosis by occurrence of death.

Of all patients, 10 (10.3%) were diagnosed with hyperleukocytosis. Among them, there were significantly more men than women compared to the group without hyperleukocytosis (90% vs. 10%; *p* = 0.012; Figure 1a). This group was also significantly older than patients without hyperleukocytosis (median, 119 vs. 76 months, respectively; *p* = 0.0011). A significantly higher percentage of leukemia with the T-ALL phenotype was diagnosed in patients with hyperleukocytosis than without (60% vs. 18.4%, *p* = 0.01; Figure 1b). Patients with hyperleukocytosis had a higher mortality rate than those without (30% vs. 4.6%; *p* = 0.0217; Figure 1c). A comparison of the demographics of patients with and without hyperleukocytosis is shown in Table 2.

### 3.3. The Relationship between WBC Count and Symptoms Accompanying Patients at Diagnosis

**Figure 2:**
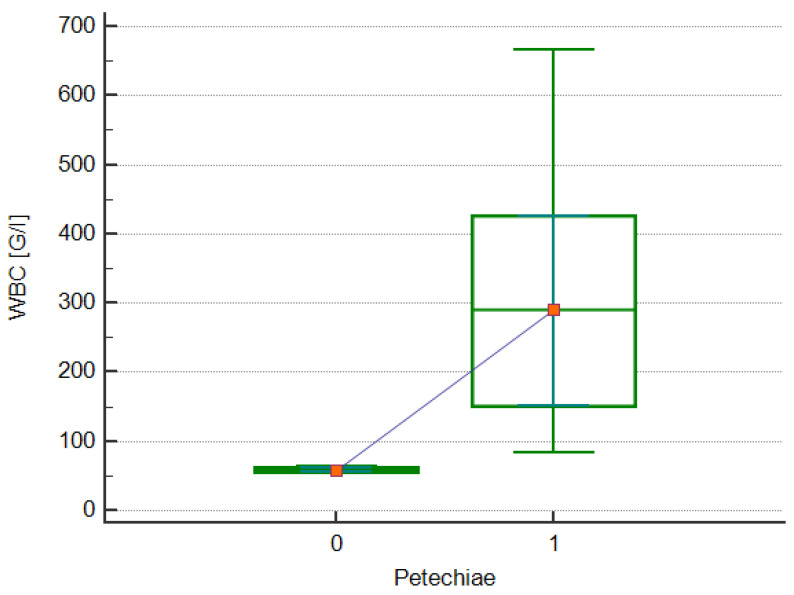
The box-whisker chart showing the relationship between petechiae occurrence (1), absence (0) and white blood cell (WBC) counts in patients with hyperleukocytosis.

**Figure 3:**
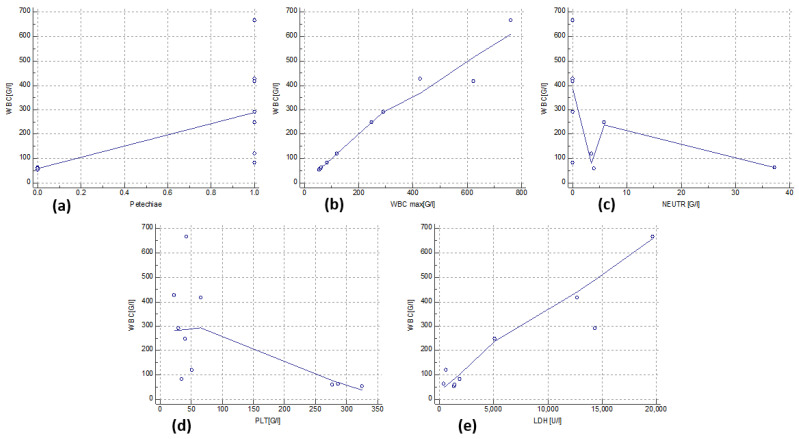
(**a**) Correlation between petechiae occurrence and white blood cell (WBC) counts in patients with hyperleukocytosis; (**b**) correlation between maximal white blood cell (WBC) counts and white blood cell (WBC) counts at the moment of admission in patients with hyperleukocytosis; (**c**) correlation between neutrophil (NEUTR) counts and white blood cell (WBC) counts in patients with hyperleukocytosis; (**d**) correlation between platelet counts (PLT) and white blood cell (WBC) counts in patients with hyperleukocytosis; and (**e**) correlation between lactate dehydrogenase (LDH) level and white blood cell (WBC) counts in patients with hyperleukocytosis.

Of the symptoms accompanying patients with hyperleukocytosis at the time of ALL diagnosis, a statistically significant correlation with WBC levels was noted only for the frequency of petechiae (median, 290.76 vs. 58.21 × 10^3^/μl, respectively; *p* = 0.0167; Figure 2). A strong positive correlation was also observed between these variables (rho = 0.798; *p* = 0.0057; Figure 3a). In addition, a significant strong positive correlation was observed between WBC levels at diagnosis and the maximum WBC levels during treatment (rho = 0.988; *p* < 0.0001; Figure 3b) and LDH activity (rho = 0.800; *p* = 0.0096; Figure 3c). Moreover, a significant moderate negative correlation was noted between the WBC level at diagnosis, the neutrophil count (rho = −0.676, *p* = 0.0458; Figure 3d), and platelet count (rho = −0.661; *p* = 0.0376; Figure 3e) at diagnosis. Leukostasis appeared in three children with hyperleukocytosis (30%), but no correlation was observed between its occurrence and the WBC level. The relationship between WBC levels, clinical symptoms, and the presentation of laboratory results in patients with hyperleukocytosis at diagnosis are shown in Table 3. The correlation between the indicated data is shown in Table 4. Moreover, a genetic test was performed at the time of diagnosis. Genetic changes were detected in three patients with hyperleukocytosis. In the first test, it was *ETV6/RUNX1*; in the second, it was *KMT2A/AFF1*; and in the third, the changes included *ETV6/RUNX1*, *TCRVG9*, and *IGHV3*.

### 3.4. The Relationship between WBC Count and Patients’ Treatment and Outcomes

**Figure 4:**
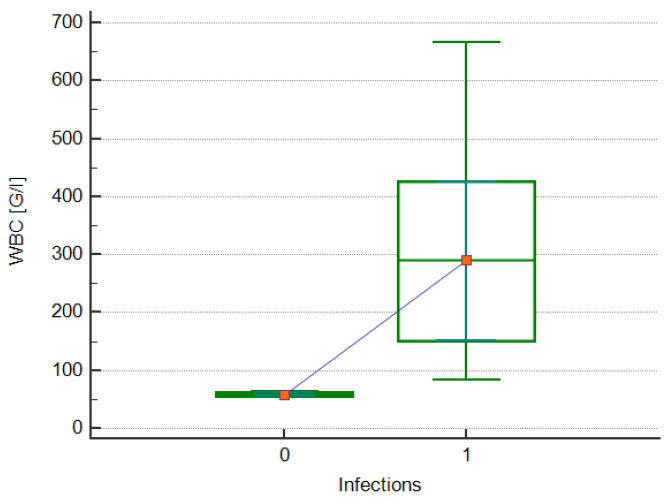
A box chart showing the relationship between the presence (1) and absence (0) of infections and white blood cell (WBC) counts in patients with hyperleukocytosis.

**Figure 5:**
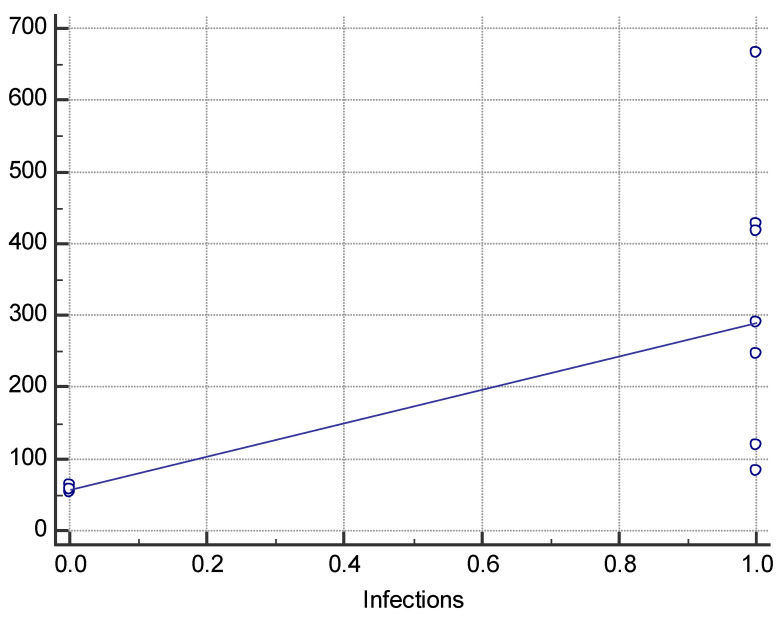
Correlation between white blood cell (WBC) counts and the occurrence of infections in patients with hyperleukocytosis.

Nearly all (80%) patients with hyperleukocytosis received standard ALL treatment according to the AIEOP-BFM ALL 2017 protocol. One patient with T-ALL received treatment according to the ALL IC-BFM 2009 regimen. One patient died before treatment was implemented. A statistically significant relationship was observed between the WBC level at the time of ALL diagnosis and the incidence of infection as a complication of treatment (290.76 vs. 58.21 × 10^3^/μL; *p* = 0.0167; Figure 4). In addition, there was a significant moderate positive correlation between the above study variables (rho = 0.798; *p* = 0.0057; Figure 5). The relationship between the WBC level at diagnosis and the course and outcome of patients with hyperleukocytosis is shown in Table 5. The correlation between the above-studied variables is shown in Table 6.

## 4. Discussion

Hyperleukocytosis (HL) is most common in patients with acute lymphoblastic leukemia (ALL) and slightly less common in patients with acute myeloid leukemia (AML) [5]. It is also sometimes observed among patients with chronic myeloid leukemia (CML) and chronic myelomonocytic leukemia (CMML). Although the criteria assume the occurrence of HL complications at a peripheral blood WBC level of more than 100,000/mm^3^, there are cases described in the literature of the appearance of HL symptoms at a WBC count as high as 50 × 10^3^, as shown in our study group. The importance of early diagnosis and the treatment of HL is its impact on mortality, which increases by 40% each week [5]. Among the causes of mortality associated with HL and hemorrhagic complications are listed first, followed by leukostasis, sepsis, respiratory failure, and renal failure, as well as disseminated intravascular coagulation (DIC) and tumor lysis syndrome (TLS) together with their consequences. For this reason, HL is considered an oncologic emergency that requires close observation and appropriate interventions [5,8]. Among the patients we described, the causes of death, in addition to ALL progression, were concomitant sepsis and CNS hemorrhage.

The molecular processes leading to HL have not yet been fully identified. The basis for the development of HL is seen in interactions between leukemic blasts and endothelial cells, which can promote leukostasis and DIC. Leukemic blasts release inflammatory cytokines such as interleukin-1β (IL-1β) and tumor necrosis factor-α (TNF-α), which increase the expression of members of the selectin family (L-, P- and E-selectins) on endothelial cells. Adhesion molecules, such as CD43, CD44, and P-selectin glycoprotein-1 ligand (PSGL-1), are present in leukocytes. The integration between these molecules promotes the adhesion of blasts to endothelial cells, causes them to roll along the endothelium, and, thus, allows them to migrate through the vessel wall into interstitial space. These molecules also affect the survival of leukemic stem cells (LSCs) and their resistance to chemotherapy. The processes in the bone marrow microenvironment that led to the massive migration of leukemic blasts into the peripheral blood are also increasingly well-understood [8].

In the literature, one can find reports on the influence of genetic abnormalities on the development of HL in ALL. One such anomaly is the presence of the *KMT2A* rearrangement in ALL, which is an independent and unfavorable prognostic factor, as it is characteristically associated with HL with relatively frequent central nervous system (CNS) involvement and an aggressive course with early relapses and poor prognosis. As a result of the occurrence of such a rearrangement, long-term survival rates in all age groups are less than 60%. One of our patients had the *KMT2A*/*AFF1* t(4;11) rearrangement. *AFF1* is described as one of the most common *KMT2A* fusion partners, along with *MLLT1* and *MLLT3* [9]. Although protocol-compliant treatment was implemented in the aforementioned patient, the prognosis was inauspicious, and death occurred within a year of diagnosis due to disease progression.

HL is sometimes associated with a number of demographic–clinical conditions of patients with ALL, as well as with effects due to the course and outcome of treatment. The results we observed in our study were the same as some results obtained by other researchers. According to Kittivisuit et al., patients with HL at diagnosis were older (91 vs. 52 months; *p* = 0.013), and the T-cell subtype of leukemia was more common among them (31.2% vs. 9.4%; *p* = 0.04). As in our study, they observed a higher mortality rate in the group with HL than without it (66.3% vs. 38.2%; *p* < 0.001) [3]. Interestingly, they also observed a significantly lower platelet count in those with HL than without it. In our study, we did not compare this parameter in the two groups, but we observed a significant negative correlation between WBC and platelet counts. Kong et al. also noted a higher incidence of HL among boys and patients with T-ALL (*p* = 0.002, *p* = 0.049, respectively), although this was only true for extreme HL above 200 × 10^3^/μl leukocytes [10]. Eguiguren et al. observed that HL was associated, as in our study, with the T-ALL subtype and elevated LDH activity (*p* < 0.0001; *p* < 0.0001, respectively) in addition to an age <1 year, mediastinal mass, and hepatomegaly. The strongest association was with HL and subtype T leukemia [12]. Nguyen et al. noted a significant association between HL and the male gender, the T-cell immunophenotype, and increased LDH activity (*p* = 0.0336; *p* < 0.0001; *p* < 0.0001, respectively), as well as increased uric acid levels, central nervous system involvement and age <1 or >9 years [13]. In addition, Alfina et al. noted an association between WBC levels with T-ALL and male gender and higher thrombocyte count [14]. Each of the aforementioned factors (age, gender, immunophenotype) associated with HL may be an adverse prognostic factor [3]. Moreover, according to some, the mere presence of HL may indicate an unfavorable prognosis for the patient, especially in the case of T-ALL [14,15,16]. Oymak et al. noted that HL is associated with a higher incidence of death, which, in the case of ALL, is due to infections (sepsis and pneumonia) [17]. HL is also associated with complications occurring during treatment and primarily induction [10]. They even affect more than 50% of patients [18]. The main complications are hemorrhage into the central nervous system, tumor lysis syndrome with hyperkalemia as a key metabolic complication, and respiratory failure [10,13]. None of the aforementioned complications were significantly associated with the WBC count in our study group. Moreover, a patient’s HL may be associated with a worse prognosis and a higher rate of recurrence. This is especially true for children with WBC > 200 × 10^3^/mm^3^, that is, with so-called extreme HL. Park et al. observed that the 10-year event-free survival (EFS) rate was significantly lower in the group with extreme HL at the baseline than in those without it (65.7% vs. 91.2%; *p* = 0.011). Moreover, patients with HL experienced recurrence in 27.2% [18]. Kittivisuit et al. noted that HL patients had significantly lower EFS and overall survival (OS) rates than those without HL (33.7% vs. 59.1%; *p* < 0.0001; 37.2% vs. 67.8%; *p* < 0.0001, respectively). In addition, they found that age <1 and >10 years, male gender, and WBC count were adverse factors associated with decreased OS in patients with HL [3]. Similar observations were made by Alfina et al., noting a lower 2-year EFS (45% vs. 68%; *p* = 0.003) in patients with HL. However, they observed no statistically significant difference in OS values in the two groups (68% vs. 77%; *p* = 0.16). According to these researchers, none of the above factors significantly affected the values of the above indicators [14].

The treatment of hyperleukocytosis may include the initiation of induction chemotherapy, intensive hydration, urine alkalinization, hydroxyurea administration, leukapheresis, or exchange transfusion [8,19]. The latter two methods contribute to cytoreduction by 30–60% and 85%, respectively [20]. They are recommended primarily as the second-line treatment for patients with symptomatic HL who cannot immediately start chemotherapy (category II of recommendations) [21,22,23,24]. Regarding the WBC cut-off threshold for initiating leukapheresis, as the prophylaxis of leukostasis (regardless of the symptoms), the guidelines indicate a count of >100 × 10^3^/mm^3^ for AML (even >50 × 10^3^/mm^3^ for monocytic subtypes of AML) and >400 × 10^3^/mm^3^ for ALL (category III of recommendations) [24,25]. Contraindications to this procedure include the severe general condition of the patient (which can be determined under careful observation in the ICU), a hemoglobin level <6 g/dL, platelet count <30 × 10^3^/mm^3^, as well as acute promyelocytic leukemia due to a high risk of bleeding [5,24,25]. The primary treatment is to start chemotherapy as soon as possible, appropriate to the final diagnosis and local protocols [8,19,20]. It should be remembered that chemotherapy is the only treatment method, and leukapheresis is only supportive [24,25,26]. It helps to reduce the WBC count and, thus, resolves the symptoms of leukostasis and, according to some authors, even improves the early death rate [27].

The limitations of our study include primarily the small size of the study group and the single-center nature of the study. Moreover, the patients did not have any comorbidities that could be clearly recorded in the documentation. The obtained results, therefore, provide a good foundation for further multi-center studies with a larger study group.

## 5. Conclusions

Hyperleukocytosis is significantly more common in older boys diagnosed with T-ALL. It is significantly associated with higher patient mortality. The WBC count in patients with HL is correlated with some clinical and laboratory manifestations (petechiae, reduced platelet levels, elevated LDH activity, and reduced neutrophil count). Higher leukocytosis is also correlated with a higher incidence of infection as a complication of ALL therapy. Our results are consistent with those obtained by other researchers.

## Figures and Tables

**Table 1:** General characteristics of the group.

Variable	N (%) or Median (IQR) [Min–Max]
Sex	
Women	51 (52.6%)
Men	46 (47.4%)
Age of diagnosis (months)	78 (45–143.25) [5–180]
Age of diagnosis (years)	6.5 (3.75–11.94) [0.42–15]
Diagnosis	
B-ALL	75 (77.3%)
T-ALL	22 (22.7%)
Death	
Yes	7 (7.2%)
No	90 (92.8%)

ALL—acute lymphoblastic leukemia, IQR—interquartile range, and N—number.

**Table 2:** Comparison of demographics of patients with and without hyperleukocytosis.

Variable	Hyperleukocytosis (*n* = 10) N (%) or Median (IQR) [Min–Max]	Non-Hyperleukocytosis (*n* = 87) N (%) or Median (IQR) [Min–Max]	*p*
Sex			0.0120 *
Women	1 (10%)	50 (57.5%)	
Men	9 (90%)	37 (42.5%)	
Age of diagnosis (months)	119 (36–157) [7–180]	76 (45–136.75) [5–179]	0.0011 *
Diagnosis			0.0100 *
B-ALL	4 (40%)	71 (81.6%)	
T-ALL	6 (60%)	16 (18.4%)	
Death			0.0217 *
Yes	3 (30%)	4 (4.6%)	
No	7 (70%)	83 (95.4%)	

ALL—acute lymphoblastic leukemia, IQR—interquartile range, and N—number. *—statistically significant results.

**Table 3:** The relationship between WBC count, clinical features and presentation of results from laboratory findings in patients at diagnosis.

CLINICAL FEATURES			
Variable	N (%)	WBC [×10^3^/μL] Median (IQR) [Min–Max]	*p*
Fever			0.5224
Yes	6 (60%)	183.27 (82.75–471.45) [62.73–666.83]	
No	4 (40%)	174.48 (56.1–359.03) [54–427.3]	
Petechiae			0.0167 *
Yes	7 (70%)	290.76 (151.35–424.84) [82.75–666.83]	
No	3 (30%)	58.21 (55.05–61.6) [54–62.73]	
Emesis			0.8311
Yes	4 (40%)	183.27 (101.1–332.28) [82.75–417.45]	
No	6 (60%)	176.75 (58.21–427.3) [54–666.83]	
Myalgia			0.6015
Yes	1 (10%)	82.75	
No	9 (90%)	247.11 (61.6–419.91) [54–666.83]	
Lymphadenopathy			0.8311
Yes	6 (60%)	205.1 (62.73–417.45) [54–666.83]	
No	4 (40%)	164.93 (70.48–337.21) [58.21–427.3]	
Hepatosplenomegaly			0.3840
Yes	9 (90%)	247.11 (76.61–419.93) [54–666.83]	
No	1 (10%)	62.73	
Mediastinal tumor			0.2100
Yes	3 (30%)	62.73 (56.18–233.75) [54–290.76]	
No	7 (70%)	247.11 (91.2–424.83) [58.21–666.83]	
Leukostasis			0.3505
Yes	3 (30%)	417.45 (289.65–604.45) [247.11–666.83]	
No	7 (70%)	82.75 (59.34–247.93) [54–427.33]	
CNS involvement			0.3505
Yes	5 (50%)	247.11 (110.26–322.43) [82.75–417.45]	
No	5 (50%)	62.75 (57.15–487.18) [54–666.83]	
Diagnosis			0.6698
T-ALL	6 (60%)	268.93 (62.73–417.45) [54–666.83]	
B-ALL	4 (40%)	101.09 (70.48–273.37) [58.21–427.3]	
**LABORATORY FINDINGS**			
**Variable**	**Median (IQR) [min–max]**		***p***
**Peripheral blood**			
WBC at diagnosis [×10^3^/μL]	183.27 (62.75–417.45) [54–666.83]		-
WBC maximal [×10^3^/μL]	183.27 (62.75–427.3) [54–760.1]		0.2313
LYM [×10^3^/μL]	67.6 (64.5–72.3) [49.1–85]		0.2303
NEUTR [×10^3^/μL]	0 (0–4.38) [0–37.2]		0.2867
RBC [×10^6^/μL]	3.81 (2.8–4.71) [0.73–5.07]		0.2313
HGB [g/dL]	9.65 (8.1–12.2) [1.8–13.3]		0.2313
PLT [×10^3^/μL]	46.5 (34–277) [22–325]		0.2313
LDH [U/L]	1874 (1167.5–13,143.25) [426–19,687]		0.4334
**Bone marrow**			
Blasts [%]	82 (73.5–88) [32–92]		0.3610
Monocytes [%]	1 (0–2.25) [0–7]		0.2331
Lymphocytes [%]	10 (5.75–15.5) [0–20]		0.4158
Band cells [%]	2 (0–2) [0–6]		0.2635
Segmented granulocytes [%]	3 (2.75–11.25) [1–78]		0.3354

N—number, WBC—white blood cells, IQR—interquartile range, ALL—acute lymphoblastic leukemia, CNS—central nervous system, LYM—lymphocytes, NEUTR—neutrophiles, RBC—red blood cells, HGB—hemoglobin, PLT—platelets, LDH—lactate dehydrogenase, and *—statistically significant results.

**Table 4:** Correlation between laboratory and clinical findings and WBC count at diagnosis.

Variable	rho	*p*
Fever	0.213	0.5543
Petechiae	0.798	0.0057 *
Emesis	0.535	0.1114
Myalgia	−0.174	0.6305
Lymphadenopathy	0.071	0.8453
Hepatosplenomegaly	0.290	0.4161
Mediastinal tumor	−0.418	0.2295
Leukostasis	0.570	0.0855
CNS involvement	0.174	0.6305
WBC max [×10^3^/μL]	0.988	<0.0001 *
LYM [×10^3^/μL]	0.150	0.7001
NEUTR [×10^3^/μL]	−0.676	0.0458 *
RBC [×10^6^/μL]	−0.188	0.6032
HGB [g/dL]	−0.303	0.9338
PLT [×10^3^/μL]	−0.661	0.0376 *
LDH [U/L]	0.800	0.0096 *
Blasts [%]	0.298	0.4032

CNS—central nervous system, WBC—white blood cells, LYM—lymphocytes, NEUTR—neutrophiles, RBC—red blood cells, HGB—hemoglobin, PLT—platelets, LDH—lactate dehydrogenase, *—statistically significant results.

**Table 5:** The relationship between WBC counts and patients’ treatment and outcomes.

Variable	N (%)	WBC [10^3^/μL] Median (IQR) [Min–Max]	*p*
TREATMENT AND THE RESULTS			
Leukapheresis			0.6750
Yes	2 (20%)	542.14 (475.45–666.83) [417.45–666.83]	
No	8 (80%)	101.1 (60.47–268.94) [54–427.3]	
Response to steroids			0.5688
Good	7 (70%)	119.43 (59.34–382.25) [54–666.83]	
Poor	0	-	
None	3 (30%)	290.76 (134.75–385.78) [82.75–471.45]	
15th day response			0.6951
<10%	6 (60%)	164.93 (62.73–290.76) [58.21–666.83]	
≥10%	2 (20%)	273.37 (119.43–427.3) [119.43–427.3]	
None	2 (20%)	235.73 (54–417.45) [54–417.45]	
33rd day of response			0.2318
Good	5 (50%)	82.75 (61.6–258.02) [58.21–290.76]	
Poor	3 (30%)	427.3 (196.4–606.95) [119.43–666.83]	
None	2 (20%)	235.73 (54–417.45) [54–417.45]	
**COMPLICATIONS OF TREATMENT**			
Infection			0.0167 *
Yes	7 (70%)	290.76 (151.35–424.84) [82.75–666.83]	
No	3 (30%)	58.21 (55.05–61.6) [54–62.73]	
Bacterial infection			0.1355
Yes	4 (40%)	332.28 (183.27–542.14) [119.43–666.83]	
No	6 (60%)	72.74 (58.21–290.76) [54–427.3]	
Fungal infection			0.1916
Yes	2 (20%)	359.03 (290.76–427.3) [290.76–427.3]	
No	8 (80%)	101.09 (60.47–332.28) [54–666.83]	
Bone marrow aplasia			0.5224
Yes	6 (60%)	183.27 (82.75–427.3) [58.21–666.83]	
No	4 (40%)	176.75 (58.37–354.11) [54–417.45]	
Gastrointestinal			0.3050
Yes	3 (30%)	247.11 (151.35–561.9) [119.43–666.83]	
No	7 (70%)	82.75 (59.34–385.78) [54–427.3]	
Hemorrhage			0.7940
Yes	2 (20%)	245.02 (62.73–427.3) [62.73–427.3]	
No	8 (80%)	183.27 (70.48–354.11) [54–666.83]	
Renal			0.3840
Yes	1 (10%)	62.73	
No	9 (90%)	247.11 (76.62–419.91) [54–666.83]	
**OUTCOMES**			
Remission			0.9168
Yes	5 (50%)	247.11 (61.6–384.78) [58.21–666.83]	
No	5 (50%)	119.43 (75.56–419.91) [54–427.3]	
Death			0.5688
Yes	3 (30%)	247.11 (151.35–374.87) [119.43–417.45]	
No	7 (70%)	82.75 (59.34–393.17) [54–666.83]	

N—number, IQR—interquartile range, WBC—white blood cells, and *—statistically significant results.

**Table 6:** Correlation between WBC count at diagnosis and patients’ treatment and outcomes.

Variable	rho	*p*
Leukapheresis	0.609	0.0615
Good response to steroids	−0.190	0.5992
15th day MRD	0.000	1.0000
33rd day MRD	0.487	0.1535
Bone marrow aplasia	0.213	0.5543
Infections	0.798	0.0057 *
Gastrointestinal complications	0.342	0.3336
Renal complications	−0.290	0.4161
Remission	0.035	0.9239
Death	0.190	0.5992
Time to death	0.127	0.7271

MRD—minimal residua disease; *—statistically significant results.

## Data Availability

Data are contained within the article.
